# Nicotine aggravates vascular adiponectin resistance via ubiquitin-mediated adiponectin receptor degradation in diabetic Apolipoprotein E knockout mouse

**DOI:** 10.1038/s41419-021-03772-y

**Published:** 2021-05-18

**Authors:** Jia Gao, Jianghong Fan, Zhijun Meng, Rui Wang, Caihong Liu, Jing Liu, Bin Liang, Jing Wang, Yaoli Xie, Jing Zhao, Rui Guo, Jianli Zhao, Xinliang Ma, Xiangying Jiao, Jimin Cao, Yajing Wang

**Affiliations:** 1 Department of Physiology, Shanxi Medical University, Taiyuan, Shanxi 030001 China; 2 Department of Cardiology, First Hospital of Shanxi Medical University, Taiyuan, Shanxi 030001 China; 3 Department of Emergency Medicine, Thomas Jefferson University, Philadelphia, PA 19107 USA

**Keywords:** Lipid signalling, Atherosclerosis

## Abstract

There is limited and discordant evidence on the role of nicotine in diabetic vascular disease. Exacerbated endothelial cell dysregulation in smokers with diabetes is associated with the disrupted adipose function. Adipokines possess vascular protective, anti-inflammatory, and anti-diabetic properties. However, whether and how nicotine primes and aggravates diabetic vascular disorders remain uncertain. In this study, we evaluated the alteration of adiponectin (APN) level in high-fat diet (HFD) mice with nicotine (NIC) administration. The vascular pathophysiological response was evaluated with vascular ring assay. Confocal and co-immunoprecipitation analysis were applied to identify the signal interaction and transduction. These results indicated that the circulating APN level in nicotine-administrated diabetic Apolipoprotein E-deficient (ApoE^−/−^) mice was elevated in advance of 2 weeks of diabetic ApoE^−/−^ mice. NIC and NIC addition in HFD groups (NIC + HFD) reduced the vascular relaxation and signaling response to APN at 6 weeks. Mechanistically, APN receptor 1 (AdipoR1) level was decreased in NIC and further significantly reduced in NIC + HFD group at 6 weeks, while elevated suppressor of cytokine signaling 3 (SOCS3) expression was induced by NIC and further augmented in NIC + HFD group. Additionally, nicotine provoked SOCS3, degraded AdipoR1, and attenuated APN-activated ERK1/2 in the presence of high glucose and high lipid (HG/HL) in human umbilical vein endothelial cells (HUVECs). MG132 (proteasome inhibitor) administration manifested that AdipoR1 was ubiquitinated, while inhibited SOCS3 rescued the reduced AdipoR1. In summary, this study demonstrated for the first time that nicotine primed vascular APN resistance via SOCS3-mediated degradation of ubiquitinated AdipoR1, accelerating diabetic endothelial dysfunction. This discovery provides a potential therapeutic target for preventing nicotine-accelerated diabetic vascular dysfunction.

## Introduction

Nicotine is an important component of cigarettes and electronic cigarettes, which has increasingly aroused public concern about life-threatening risks^1–3^. New research shows that adults who report puffing E-cigarettes, or vaping, have strikingly higher odds of having a heart attack and coronary artery disease than non-users^4^. Besides, nicotine consumers with diabetes experience more damage to the cardiovascular system than people with diabetes alone^5^. Although nicotine consumers are rarely obese, smokers with diabetes have more severe insulin resistance than a non-smoker^6–8^. Therefore, the body weight and conventional body mass index (BMI) are inadequate to interpret diabetes in people who smoke^9,10^.

There is a bulk of evidence that lipid uptake is not related to nicotine-induced insulin resistance on smokers with type 2 diabetes^11^, thus it is necessary to further study the potential molecular mechanisms leading to the persistence of insulin resistance. In addition, nicotine cessation does not stop but promotes vascular disease development secondary to diabetes^12,13^. Whether and how nicotine-induced cardiovascular system disorder leads to and exaggerates diabetic vasculopathy needs further clarification. Adiponectin (APN), secreted from adipocytes, is an adipokine whose expression and plasma levels are inversely related to obesity and insulin resistance states^14^.

Therefore, this study aimed to access whether nicotine causes and accelerates vasculopathy in diabetes and if so, to investigate the mechanisms of nicotine-induced diabetic vasculopathy.

## Material and methods

### Animal model

The animal study protocol has been approved by the institutional animal care and used committees (IACUC) of Shanxi Medical University. The experimental procedures were in adherence with the National Institutes of Health Guidelines on the Use of Laboratory Animals. The adult Apolipoprotein E knockout (ApoE^−/−^) mice (C57BL/6J background) were randomly divided into four groups: normal diet (ND)-treated group; high-fat diet (HFD, 60 kcal% fat, Cat #D12492, Research Diets, New Brunswick, NJ, USA)-treated group; nicotine (NIC, 1.5 mg/kg/d through osmotic minipumps)/ND-treated group and NIC + HFD-treated group (NIC + HFD). Nicotine was purchased from Sigma-Aldrich (N-008, St. Louis, MO, USA). Sample size was pre-determined and no blinding was used. All groups were continuously fed with ND or HFD for 12 weeks. Exclusion criteria of mice: infection or death. Mice were fasted overnight and body weight, plasma biochemical characteristics including total cholesterol (TC), triglycerides (TG), glucose, insulin, and APN levels were determined.

At the end of 6 and 12 weeks, the aortic segment from the heart to iliac bifurcation was excised and cleaned, removing adherent tissues. Vascular segments were either homogenized for the immunoblotting assay or cut into 2–3 mm vascular segments for vascular function determination.

### Aortic contractility and relaxation assay

The mouse aortic segment was quickly removed and placed in an ice-cold Krebs-Henseleit (K-H) buffer consisting of: KCl (1.752 mM), KH_2_PO_4_ (0.8165 mM), MgSO_4_•7H_2_O (1.479 mM), NaCl (34.48 mM), NaHCO_3_ (1.05 mM), Dextrose (0.99 mM), and CaCl_2_•2H2O (0.1867 mM). Then, 2–3 mm aorta rings were isometrically mounted upon a Multi-Wire Myograph System (DMT610M, Aarhus, Denmark). The vessel rings were exposed to K-H buffer in the tissue bath and K-H buffer was replaced every 15 min. After equilibration and preloading to 4 mN, 50 μl norepinephrine (10^−9^ to 10^−5^ M) and 50 μl acetylcholine (Ach, an endothelium-dependent vasodilator, 10^−9^ to 10^−4^ M) were used to evaluate vessel contractibility response. After achieving balanced states, globular adiponectin (gAPN, 0.3, 1, 3, and 10 μg/ml) was administered to determine APN-induced vasorelaxation response. Globular APN was obtained from Aviscera Bioscience (Shanghai, China). After 1 h equilibration, acidified NaNO_2_ (an endothelium-independent vasodilator, 10^−9^ to 10^−4^ μM, prepared by dissolving NaNO_2_ in 0.1 N HCl titrated to pH 2.0) was then introduced. Data were collected and analyzed by a Power lab 8.0 system (ADInstruments, NSW, Australia).

### Biochemistry and enzyme-linked immunosorbent assay (ELISA)

Serum samples were collected from ApoE^−/−^ mice once a week during the treatment with nicotine and HFD for 12 weeks. We measured fasting blood glucose (FBG), TC, and TG on AU5800 chemistry Analyzer (Beckman Coulter, Shanghai, China). Total APN were determined by mouse APN ELISA kits (R&D systems, Cat #PMRP300, Minneapolis, MN, USA). The insulin levels were determined by mouse insulin ELISA kits (ALPCO, Cat #80-INSMS-E01, Salem, NH, USA) following the manufacturer’s instructions.

### RNA extraction and real-time PCR

Total RNA was isolated using total RNA extraction and purification kit (Sangon Biotech, Shanghai, China) and then RNA concentration and purity were determined at A260/A280 on a micro spectrophotometer (Nano-100, Eppendorf, Shanghai, China). Real-time PCR was conducted with PrimeScript™ RT reagent Kit with gDNA Eraser (Takara, Dalian, China). Subsequently, the relative mRNA levels were assessed using the 2^−ΔΔCt^ method.

### Cell culture

Human umbilical vein endothelial cells (HUVECs) were purchased from the China Academy of Science (Shanghai, China). All cell lines were authenticated by STR profiling and routinely tested for Mycoplasma contamination. Fetal bovine serum was obtained from CellMax (Beijing, China). All other cell culture-related reagents were purchased from Boster Biological Technology (Wuhan, China). HUVECs were cultured in endothelial growth medium containing 10% fetal bovine serum, 2 mM glutamine, 100 U/ml penicillin, and 100 µg/ml streptomycin. Cells were randomized to receive one of the following treatments after reaching 80% confluence: control group (normal glucose/normal lipid, NG/NL, the normal glucose contains 5.5 mM D-glucose + 19.5 mM L-glucose); high glucose/high lipid group (HG/HL, 25 mM D-glucose/200 μM palmitate, 24 h incubation); nicotine group (10^-7^ M, 24 h incubation), and NIC + HG/HL group. MG132 (a peptide aldehyde, potent cell-permeable proteasome inhibitor, 10 µM, 6 h) was administrated to HUVECs followed with NIC or HG/HL treatment. MG-132 was purchased from APExBIO (Cat#A2585, Houston, TX, USA).

### Western blotting

50 μg total proteins per sample were separated by gel electrophoresis and transferred to polyvinylidene fluoride (PVDF) membranes followed by blocking with 5% skimmed milk at room temperature for 1 h. Then the membranes were incubated with the appropriate primary antibodies against APN receptor 1 (AdipoR1; 1:1000 dilution, bs-0610R, Bioss, USA), suppressor of cytokine signaling 3 (SOCS3; 1:200 dilution, sc-518020, Santa Cruz, TX, USA), ERK1/2 (1:1000 dilution, #8544, Cell Signaling Technology, MA, USA), Ubiquitin (1:200 dilution, P4D1, Santa Cruz, TX, USA), and β-Actin (1:1000 dilution, BM3873, Boster, Shanghai, China) at 4 °C overnight. After washing with TBST three times, the membranes were incubated with the secondary HRP-conjugated antibody (anti-mouse or anti-rabbit antibody, 1:10,000 dilution, Boster, Shanghai, China) for 1 h. The image was captured on ChemiDoc MP Imaging System (Bio-Rad, CA, USA) and the density was quantified by ImageJ (NIH).

### Immunofluorescence staining assay

To detect the distribution of AdipoR1 and SOCS3 in HUVECs, the cells after the treatment were fixed with 4% paraformaldehyde for 15 min, penetrated by 0.5% Triton X-100 for 20 min, and then blocked with goat serum. Then the cells were incubated with anti-AdipoR1 (1:200, bs-0610R, Bioss, Beijing, China) and anti-SOCS3 (1:200, sc-518020, Santa Cruz, TX, USA) antibody at 4 °C overnight. After balanced to room temperature, cells were incubated with corresponding secondary antibody (1:100, Boster, Shanghai, China) in the dark for 1 h. 4ʹ,6-diamidino-2-phenylindole (DAPI, Boster, Shanghai, China) was used to stain the nuclei for 5 min. The cells were imaged under a laser scanning confocal microscope (FV300, Olympus, Tokyo, Japan).

### Small interfering RNA transfection

The siRNA-SOCS3 (5ʹ-CCACAAGTGGATTCTCCTTdTdT-3ʹ) and the non-target negative control siRNA (Scramble) were purchased from Qiagen. Transfection was performed using the HiPerFect transfection reagent according to the manufacturer’s protocol. After 6–8 h of transfection (final concentration 100 nM), the medium was replaced by the fresh normal medium.

### Co-immunoprecipitation

After treatment, HUVECs were lysed and homogenized. The protein content of lysed cells was determined by BCA method. Cell samples (1 mg/sample) were pulled down with 2 μg anti-AdipoR1 antibody followed by the addition of 20 μl protein A + G agarose beads. Non-immune rabbit IgG served as the negative control. The beads were extensively washed with lysis buffer and proteins were eluted by elution buffer and proceeded to western blot analysis.

### Statistical analysis

All numerical data were presented as mean ± SD. Statistical analysis between two groups was analyzed by Student’s *t* test, comparison of three or more groups using one-way analysis of variance (ANOVA) or two-way ANOVA followed by Tukey’s for post hoc *t* test. Normality of data was assessed via a Shapiro-Wilk normality test. Homogeneity of variance test was performed. *P* < 0.05 was considered statistically significant. Each experiment was replicated multiple time (>6). All statistical analyses were performed via GraphPad Prism 9.0.

## Results

### Nicotine induced vascular APN resistance and accelerated in ApoE^−/−^ HFD mice

Adiponectin (APN) is a novel adipokine regarded as “adipocyte-derived insulin”^15^ and it is an essential molecule maintaining insulin responsiveness through activating AMPK and PPARα^16,17^. Since APN exerted anti-diabetic and cardiovascular protective role^18–21^, and nicotine decreased plasma APN level^22,23^, we speculated that APN may be the link between nicotine and the accelerated diabetic vascular complications.

**Fig. 1:**
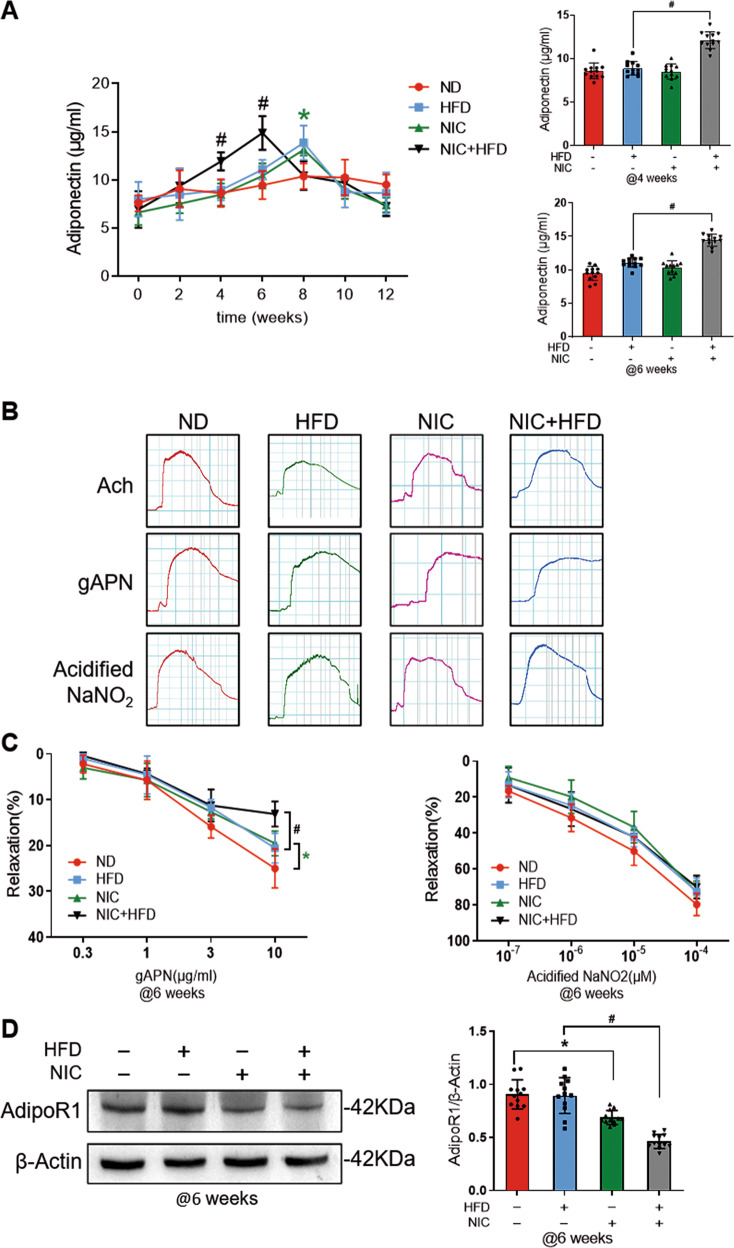
Nicotine induced and accelerated vascular APN resistance in HFD mice. **A** Circulation APN level was elevated at 4 weeks and peaked at 6 weeks after nicotine treatment on HFD mice. *n* = 12 animals/group. ^*^P < 0.05 vs. ND group; ^#^*P* < 0.05 vs. HFD group. Data were analyzed by two-way ANOVA. **B**, **C**. Vascular ring assay exhibited that gAPN-induced vasodilatation was significantly reduced in nicotine-treated HFD groups compared with the HFD group. gAPN-induced vasodilatation was reduced in nicotine-treated 6 weeks group compared with the ND group. Concentration-dependent vasorelaxation in response to acidified NaNO_2_ was normal. *n* = 12 animals/group. ^*^P < 0.05 vs. ND group; ^*#*^*P* < 0.05 vs. HFD group. Data were analyzed by two-way ANOVA. **D** The expression of AdipoR1 in vessels after nicotine and HFD treated for 6 weeks. *n* = 12 animals/group. ^*^P < 0.05 vs. ND group; ^#^*P* < 0.05 vs. HFD group. Data were analyzed by one-way ANOVA. NIC, nicotine; HFD, high-fat diet; ND, normal diet; gAPN, globular adiponectin.

Feeding with HFD for 12 weeks resulted in a dramatic increase of body weight in ApoE^−/−^ mice. Administration of nicotine significantly decreased the body weight in ND-fed mice and more pronounced in HFD-fed mice after 6 weeks (Supplementary Fig. 1A). However, glucose level was discernibly increased at 4 weeks in NIC + HFD (Supplementary Fig. 1B) compared to the HFD group. The cholesterol and triglyceride levels in NIC + HFD mice were increased at 6 weeks onward (Supplementary Fig. 1C, D) compared to the HFD group. However, the glucose, cholesterol, and triglyceride levels in NIC mice were moderately elevated at 6 weeks compared with the ND mice (Supplementary Fig. 1B–D). Concomitantly, insulin resistance was appeared at 6 weeks in NIC + HFD, whereas HFD induced insulin resistance at 8 weeks. NIC-induced insulin resistance existed at 12 weeks compared to ND group (Supplementary Fig. 1E). Notably, as illustrated in Fig. 1A, APN level was increased at 4 weeks and peaked at 6 weeks in NIC + HFD mice, 2 weeks earlier than those with HFD alone. APN level in NIC mice elevated and peaked at 8 weeks compared to the ND mice (Fig. 1A).

To determine whether nicotine-induced APN resistance impacted the vascular response, we conducted the following three serial experiments. Firstly, gAPN-induced vasodilatation was assessed on aortic vascular segments isolated from the mice treated with NIC and HFD for 6 weeks. As summarized in Fig. 1B, C, with the addition of gAPN, there was a markedly declined vasorelaxation in NIC + HFD-treated group compared with the HFD group, whilst no significant difference was detected in vasodilation induced by acidified NaNO_2_. In addition, compared with ND mice, gAPN-induced vasodilation was significantly reduced in NIC-treated mice (Fig. 1B, C).

Secondly, as it has been demonstrated that APN receptor (AdipoR) phosphorylation may involve in the regulation of APN sensitivity in multiple organs^24^, and AdipoR1 is abundantly expressed in endothelial cells; therefore, we further investigated the expression of AdipoR1 in aortic tissue. As illustrated in Fig. 1D, we found that AdipoR1 expression was obviously reduced in NIC treated alone compared with ND mice and further decreased in NIC + HFD groups compared with HFD at 6 weeks, which strongly indicates that nicotine primed the reduction of AdipoR1 level.

**Fig. 2:**
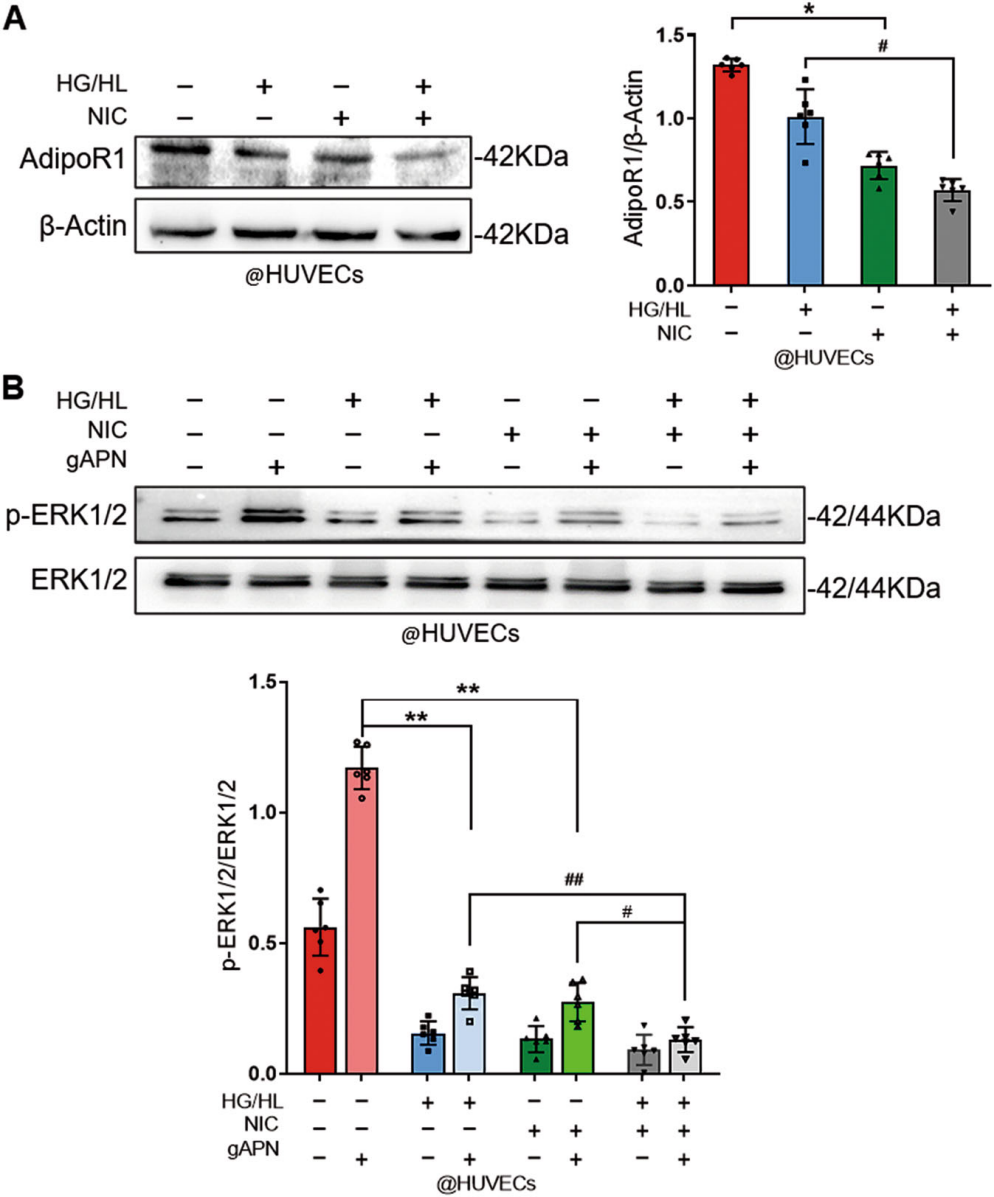
Nicotine induced and accelerated APN resistance in diabetic endothelial cells. **A** The expression of AdipoR1 in HUVECs after nicotine and HG/HL treatment. *n* = 6 dishes/group. ^*^P < 0.05 vs. control group; ^#^*P* < 0.05 vs. HG/HL group. Data were analyzed by one-way ANOVA. **B** APN-induced ERK1/2 phosphorylation was suppressed in the presence of nicotine or HG/HL and further inhibited in nicotine combined HG/HL treatment. *n* = 6 dishes/group. ^**^P < 0.01 vs. control + gAPN group; ^##^*P* < 0.01 vs. NIC + HG/HL + gAPN group. Data were analyzed by two-way ANOVA. NIC, nicotine; HFD, high-fat diet; ND, normal diet; HG/HL, high glucose/high lipid; gAPN, globular adiponectin.

Thirdly, to precisely identify whether NIC modulates AdipoR1 level in diabetic endothelial cells, HUVECs were treated with NIC in the presence of HG/HL. As shown in Fig. 2A, the expression of AdipoR1 was suppressed in NIC + HG/HL compared with HG/HL group; meanwhile, NIC treatment depressed the AdipoR1 expression compared with control. APN is an upstream molecule of the MAPK(ERK1/2) signaling axis, and MAPK(ERK1/2) phosphorylation has been well accepted as a readout of APN intracellular signaling^18^. As illustrated in Fig. 2B, APN-induced phosphorylation of ERK1/2 was significantly blunted after HUVECs were exposed to NIC (10^−7^ M). The induced ERK1/2 activation was further suppressed in NIC + HG/HL compared with HG/HL. These results suggested that nicotine blocks the APN’s signal transduction by reducing AdipoR1 level, then blunts gAPN-induced ERK1/2 activation, contributing to APN resistance, which was further aggravated in the diabetic setting.

### Nicotine augmented SOCS3’s expression, which was further deteriorated in a diabetic setting

**Fig. 3:**
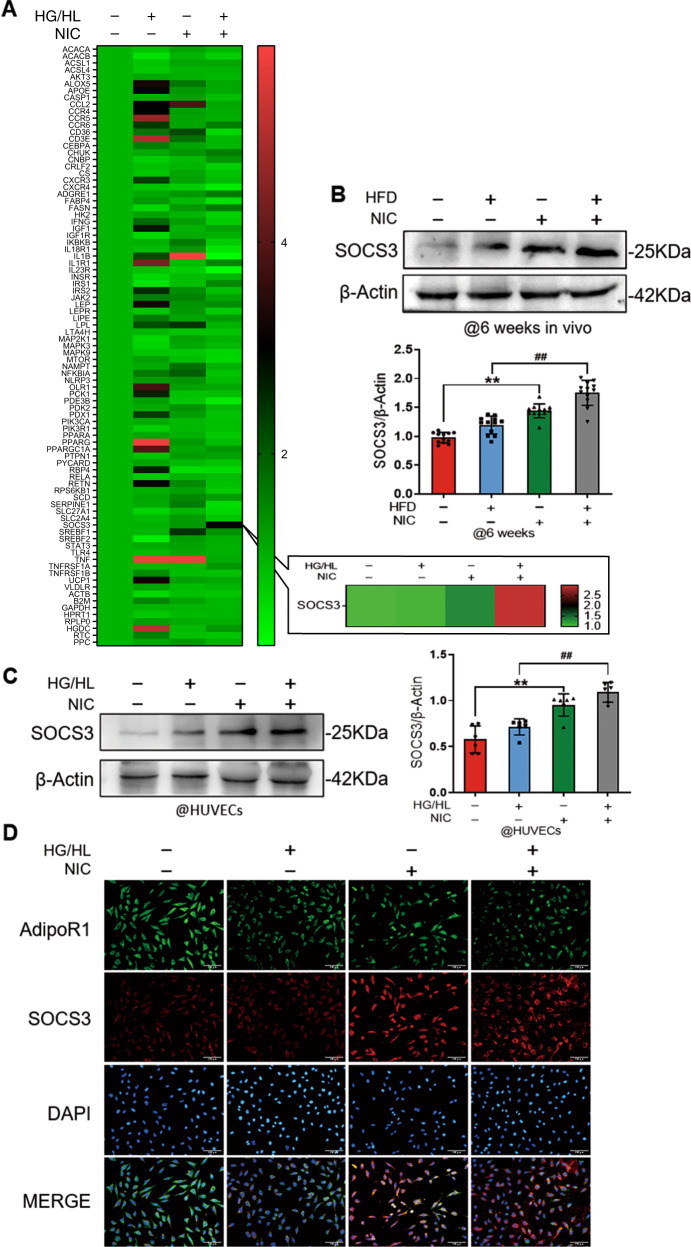
SOCS3 was upregulated by NIC and further elevated in NIC combined with HG/HL treatment. **A** RNA profile assay showed that SOCS3 was markedly induced by NIC and further augmented with HG/HL. **B** Western blot validated SOCS3 upregulation in vivo mice with NIC treatment. SOCS3 was further elevated in NIC administration on HFD mice. *n* = 12 animals/group. ^*^P < 0.05 vs. ND group; ^##^*P* < 0.01 vs. HFD group. Data were analyzed by one-way ANOVA. **C** The protein level of SOCS3 has verified in HUVECs with NIC or HG/HL treatment. *n* = 6 dishes/group. ^**^P < 0.01 vs. control group; ^##^*P* < 0.01 vs. HG/HL group. Data were analyzed by one-way ANOVA. **D** The immunofluorescence staining showed that NIC or NIC + HG/HL induced SOCS3-AdipoR1 co-localization in HUVECs (scale bar = 100 µm). NIC, nicotine; HFD, high-fat diet; ND, normal diet; HG/HL, high glucose/high lipid.

To identify how nicotine exacerbates APN resistance in diabetic endothelial cells, we conducted qPCR array on vascular biology profile panel to explore the possible molecular mechanisms responsible for APN resistance. To our surprise, among all the screened molecules, cytokine signaling 3 (SOCS3) was the only molecule significantly elevated by NIC and further increased in NIC + HG/HL (Fig. 3A). To obtain more direct evidence, we validated SOCS3 expression in aortic segments of HFD and HFD + NIC animals. We demonstrated that an increased tendency of SOCS3 expression in HFD animals, but it was markedly augmented in NIC treatment and further enhanced in NIC + HFD groups (Fig. 3B). Moreover, SOCS3 expression was significantly increased in NIC and NIC+ HG/HL-treated HUVECs (Fig. 3C). Finally, the immunofluorescence staining showed that SOCS3 was co-localized with AdipoR1 in HUVECs administrated with NIC or NIC + HG/HL, suggesting that there is an interaction between SOCS3 and AdipoR1 (Fig. 3D).

### Nicotine increased the ubiquitin-mediated degradation of AdipoR1 through elevating SOCS3 expression

SOCS3 was involved in protein ubiquitination and degradation^25^. However, whether degradation of AdipoR1 accounts for SOCS3-mediated ubiquitination and subsequent degradation. More importantly, whether this process is influenced by nicotine has not been previously investigated. To evaluate this hypothesis, serial experiments were performed.

**Fig. 4:**
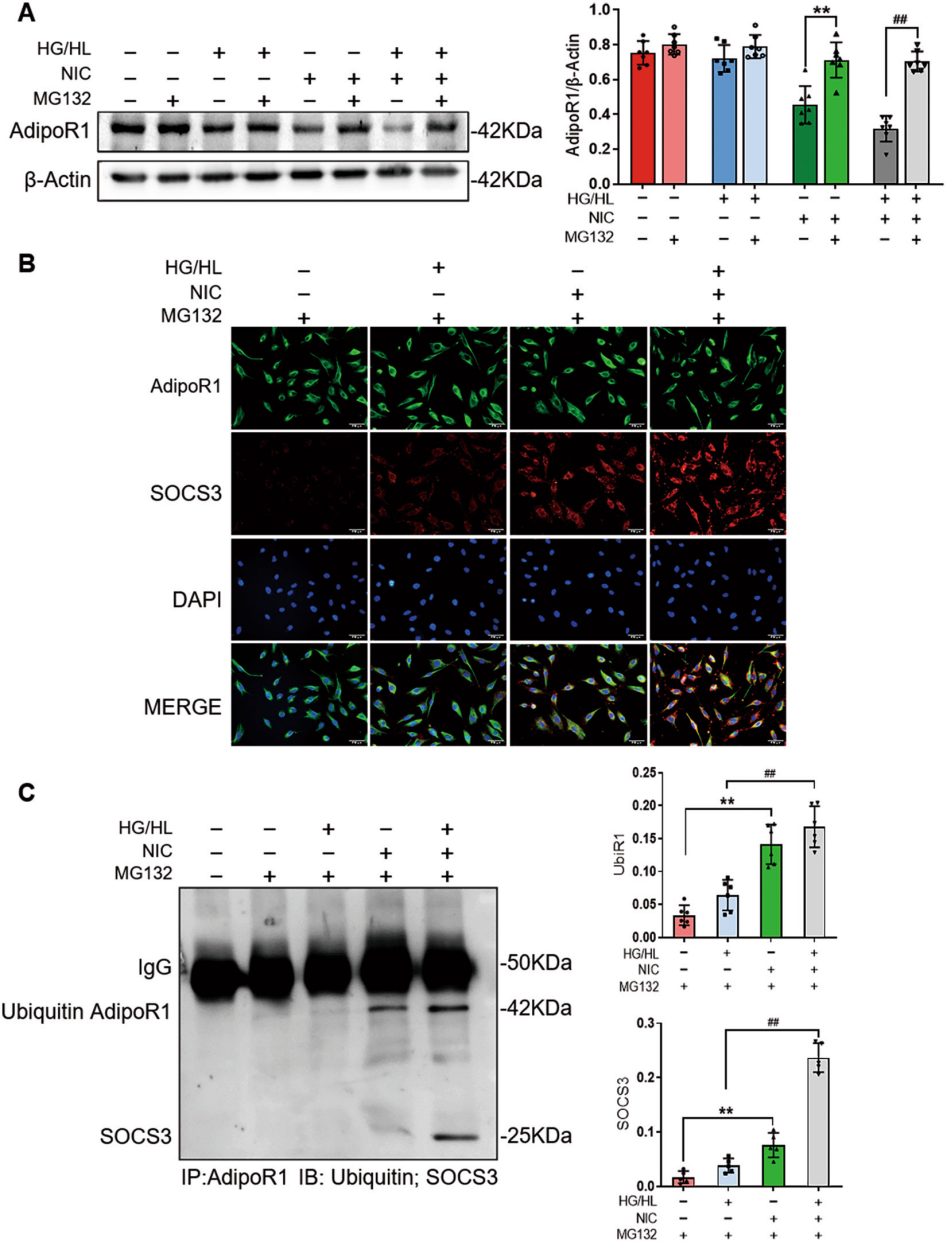
Nicotine increased the ubiquitin-mediated AdipoR1 degradation via SOCS3. **A** MG132 partially reversed NIC + HG/HL-induced AdipoR1 reduction. *n* = 6 dishes/group. ^**^P < 0.01 vs. NIC group; ^##^*P* < 0.05 vs. NIC + HG/HL group. Data were analyzed by two-way ANOVA. **B** Immunofluorescence assay showed that NIC or HG/HL-promoted AdipoR1 degradation was blocked by MG132 in HUVECs (scale bar = 50 µm). **C** The interaction between SOCS3 and ubiquitinated AdipoR1 was evidenced by co-immunoprecipitation assay. *n* = 6 dishes/group. ^**^P < 0.01 vs. control + MG132 group; ^##^*P* < 0.05 vs. HG/HL + MG132 group. Data were analyzed by one-way ANOVA. NIC, nicotine; HG/HL, high glucose/high lipid; UbiR1, ubiquitinated AdipoR1.

Firstly, MG132 was administrated to evaluate the influence of AdipoR1 degradation in HUVECs with NIC or HG/HL treatment. We identified that MG132 blocked NIC-induced AdipoR1 degradation and attenuated NIC + HG/HL-induced AdipoR1 reduction; however, AdipoR1 was not influenced by MG132 when challenged with HG/HL (Fig. 4A). These results suggested that NIC initiated AdipoR1 decline and further aggravated the decrease of AdipoR1 in HG/HL environment through ubiquitin-mediated degradation. Secondly, in order to further obtain evidence of interaction between SOCS3 and AdipoR1 in this process, we conducted the confocal co-localization analysis and co-immunoprecipitation. Confocal co-localization assay revealed that nicotine promoted the interaction between AdipoR1 and SOCS3 and reinforced their interface in the circumstance of HG/HL in endothelial cells (Fig. 4B). Meanwhile, co-immunoprecipitation assay revealed that the interaction between SOCS3 and AdipoR1 significantly increased in the presence of nicotine and was aggravated in NIC + HG/HL treatment (Fig. 4C).

### SOCS3 was required for nicotine downregulation of AdipoR1 in type 2 diabetes

**Fig. 5:**
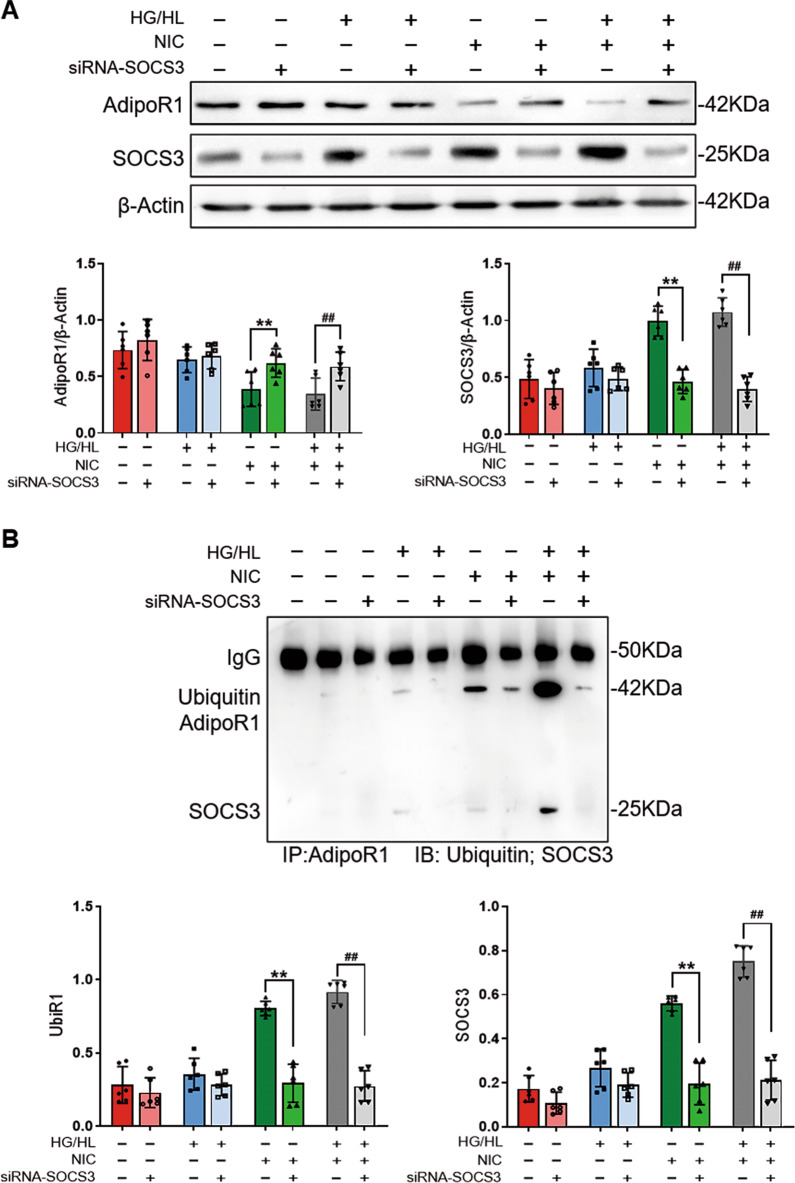
SOCS3 was required for nicotine-induced AdipoR1 downregulation in diabetes. **A** SOCS3 deficiency restored AdipoR1 level that was suppressed by nicotine or NIC + HG/HL. Top, the western blot showed the protein levels of AdipoR1 and SOCS3. β-Actin as internal references. Bottom, quantification of the density of western panel. *n* = 6 dishes/group. ^**^P < 0.01 vs. NIC group; ^##^*P* < 0.05 vs. NIC + HG/HL group. Data were analyzed by two-way ANOVA. **B** SOCS3 deficiency abolished AdipoR1 ubiquitination induced by nicotine or HG/HL. Top, co-immunoprecipitation showed the protein level of ubiquitinated AdipoR1 and SOCS3. Bottom, quantification of the density of western panel. *n* = 6 dishes/group. ^**^P < 0.01 vs. NIC group; ^##^*P* < 0.05 vs. NIC + HG/HL group. Data were analyzed by two-way ANOVA. NIC, nicotine; HG/HL, high glucose/high lipid; UbiR1, ubiquitinated AdipoR1.

To investigate whether SOCS3 is responsible for the loss of AdipoR1 and lead to APN signaling disruption, loss-of-function strategy was employed to identify SOCS3 role in the regulation of AdipoR1 level. As illustrated in Fig. 5A, NIC or NIC + HG/HL-induced AdipoR1 reduction was abolished in the SOCS3 deficiency group compared with the respective control. To further elaborate the underlying mechanism, SOCS3-deficient HUVECs was challenged by NIC or HG/HL treatment. We evaluated the AdipoR1 ubiquitination level and the results showed that AdipoR1 ubiquitination was markedly reduced in SOCS3-deficient group (Fig. 5B). Furthermore, when SOCS3 was absent, AdiopR1 and SOCS3 co-participation levels were largely reduced in the presence of NIC + HG/HL. These results indicated that SOCS3 is the key molecule to mediate AdipoR1 ubiquitinoylation and then proteosomes degrade ubiquitinated AdipoR1.

**Fig. 6:**
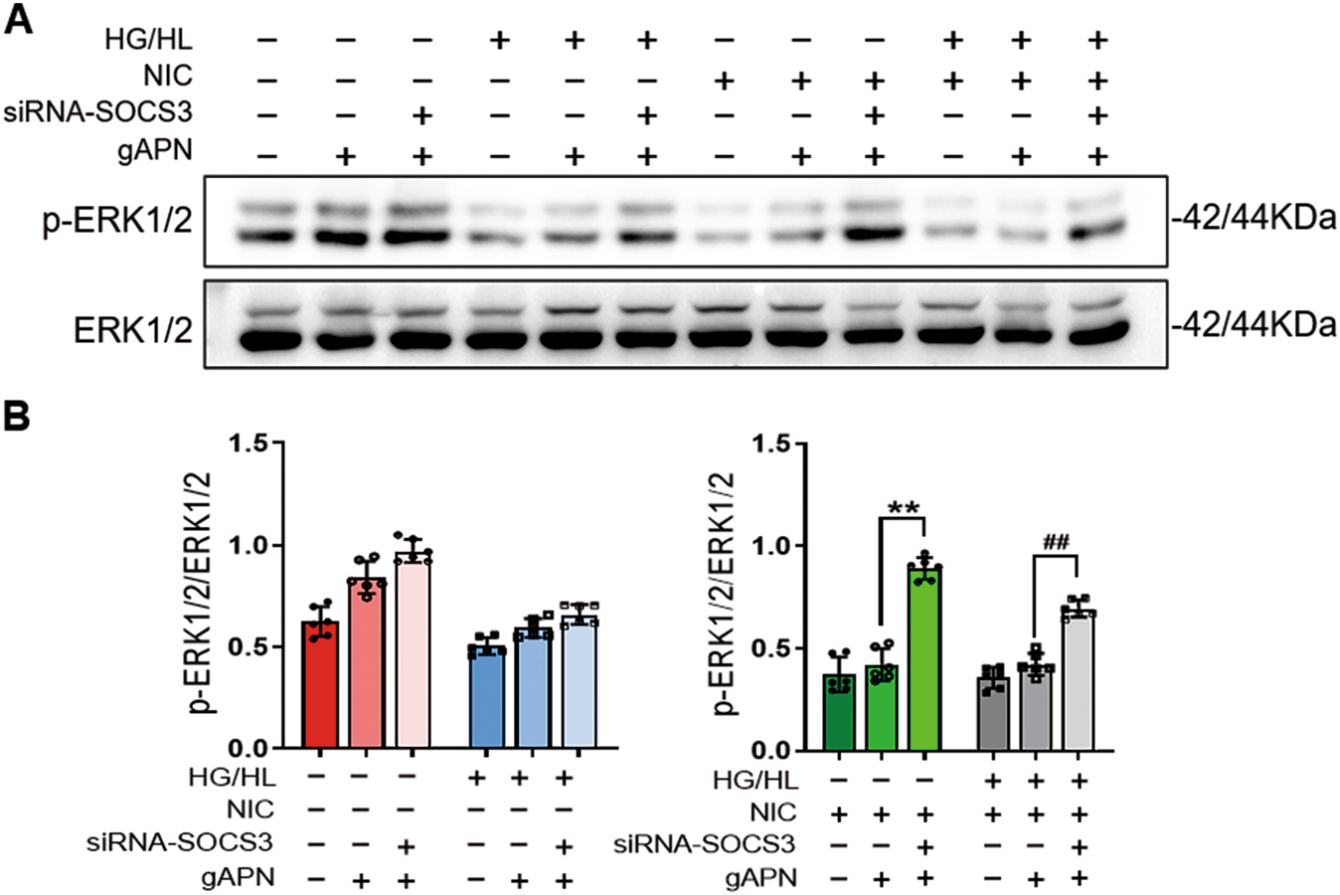
SOCS3 deficiency restored APN activation of ERK1/2 that was suppressed by NIC and NIC + HG/HL. **A** Western blot detected the level of phosphorylation of ERK1/2. **B** Quantification of the density of ERK1/2. *n* = 6 dishes/group. ^**^P < 0.01 vs. NIC + gAPN group; ^##^*P* < 0.05 vs. NIC + HG/HL + gAPN group. Data were analyzed by two-way ANOVA. NIC, nicotine; HG/HL, high glucose/high lipid; gAPN, globular adiponectin.

Finally, we evaluated the activation of ERK1/2 to further clarify whether SOCS3 deficiency restored the impaired APN signal. The results demonstrated that the inhibition of APN-induced ERK1/2 signals was prohibited in nicotine or NIC + HG/HL-administrated SOCS3-deficient HUVECs (Fig. 6A, B). These data suggested that SOCS3 is critical for nicotine-induced AdipoR1 downregulation in diabetic conditions and responsible for the loss of APN signaling response.

## Discussion

In this study, we report a discovery that nicotine induces vascular APN resistance, and further promotes the development of diabetic vascular dysfunction in the HFD-induced diabetes mice model. Nicotine upregulates E3 ligase SOCS3, which leads to AdipoR1 ubiquitination responsible for the decrease of AdipoR1. The downregulation of AdipoR1 resulted in the reduction of the signal biological response to APN, while the inhibition of SOCS3 restores the APN response. These data indicate that SOCS3 upregulated by nicotine is an important contributing factor in suppressing APN sensitivity in obesity and diabetes. Therapies aiming at inhibiting SOCS3 in the vascular system may effectively reverse diabetic vascular dysfunction and APN resistance in diabetic smokers.

In recent years, there has been a significant increase in electronic cigarettes (nicotine as main component) which was called a safer alternative to conventional cigarettes^26,27^. However, E-cigarettes not only do not improve the smoking cessation rates of traditional smokers, but also attract young people, leading to an expansion of the nicotine market^28,29^. Nicotine causes high risks in people with cardiovascular disorders, and more impairment in people with diabetes^30,31^. Diabetes accompany with hyperadiponectinemia^32^. It appears much earlier than insulin resistance^33^, which indicates it may play a causative role in the development of insulin resistance and consequent distortion of diabetic vascular response. APN resistance reduces the biologic response to APN^24,34,35^ and is related to APN receptor’s phosphorylation^36^. However, few reports on whether and how nicotine directly causes APN resistance leads to diabetic vascular dysfunction. It has been reported that APN resistance contributes to cardiovascular disease^24^ and precedes the accumulation of skeletal muscle lipids and insulin resistance in high-fat-fed rats^33^. Our findings revealed that nicotine provoked the APN resistance in the vasculature and further disturbed APN response in a diabetic setting, which was demonstrated by the decrease of vasodilation induced by APN in the diabetic ApoE^−/−^ mice. Our study chose ApoE^−/−^ mice as the research mice model. Based upon other researches and our lab’s primary results, compared with ApoE^−/−^ mouse^37^ and other mammalian models (such as non-human primates^38,39^ and swine^40^), the wild-type mouse (C57BL/6 J background) falls far short of characteristics that develop morphologically similar lesions reflecting all stages of the diabetic vascular disease when compared to those found in humans^41^. Hence, ApoE^−/−^ are the most suitable model currently for studying the diabetes-induced vascular injury.

Consistently with the previous report that the hormone resistance accounts for alteration on receptor level, we demonstrated that the APN receptor’s nicotine-provoked degradation plays an important role in APN resistance. In the present study, we further found that the vascular response to APN was declined in nicotine intervention group and SOCS3 was the key regulator in mediating the downregulation of APN receptor by ubiquitination mechanism. We also reported that the circulating APN level increased from the 4th week to peak at the 6th week, which indicated that the increase of APN is associated with AdipoR1 ubiquitination. Although we observed that nicotine significantly affects APN function in the vascular system by inducing resistance, the contradiction of body weight and abnormal lipid profiles in the circulation may be related to the adipose dysfunction caused by nicotine, inhibiting adipose expansion or proliferation.

Ubiquitin involves almost all aspects of eukaryotic biology including protein degradation, DNA repair, endocytosis, autophagy, transcription, immunity, and inflammation^42,43^. As an E3 ubiquitin ligase, SOCS3 targets associated proteins for proteasomal degradation^44^. Our study identified that nicotine upregulates SOCS3 expression, promoting the interaction between AdipoR1 and SOCS3, leading to AdipoR1 ubiquitination, whereas the SOCS3 inhibition restores AdipoR1 degradation, and reverse the corresponding AdipoR1 signals. In addition, our research shows that AdipoR1 ubiquitination hinders APN signaling, which is evidenced by the inhibition of ERK1/2 activation, a conventional signaling pathway activated by APN. However, there are still many unresolved questions that warrant further investigation. The mechanism by which nicotine increases SOCS3 is in great need to be explored.

In summary, our study identified that nicotine-provoked SOCS3-mediated AdipoR1 ubiquitination is a key mechanism of APN resistance in diabetes with nicotine. These findings help explain the general phenomenon of the accelerated development of diabetes in smokers, and may also help us understand that inhibiting AdipoR1 ubiquitination as a novel therapeutic target holds promise to prevent nicotine-induced diabetic endothelial dysfunction.

Supplementary information is available at Cell Death & Disease’s website.

## Supplementary information

Supplementary figure.

## Data Availability

The datasets used and/or analyzed during the current study are available from the corresponding author on reasonable request.
