# Supplementary figures and images for: Nicotine aggravates vascular adiponectin resistance via ubiquitin-mediated adiponectin receptor degradation in diabetic Apolipoprotein E knockout mouse
Source: Cell Death Dis. 2021 May 18;12(6):508. doi: 10.1038/s41419-021-03772-y (PMC8131622; doi:10.1038/s41419-021-03772-y)

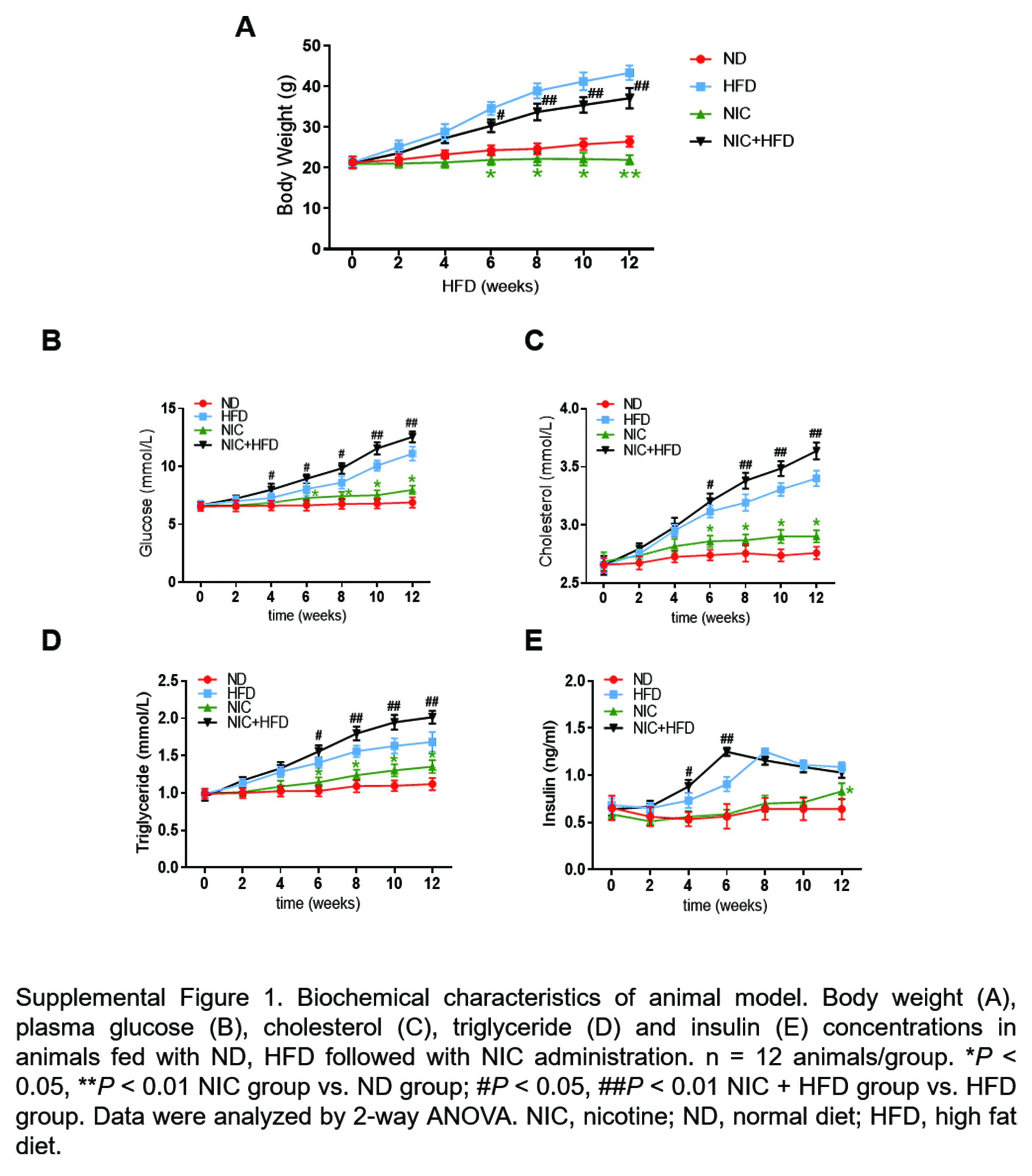

Supplement: Supplementary file 1 — Supplementary figure. [file 41419_2021_3772_MOESM1_ESM.tif]
